# Book Reviews

**Published:** 1804-03-01

**Authors:** 


### CRITICAL ANALYSIS

OF THE

### RECENT PUBLICATIONS

ON THE

### DIFFERENT BRANCHES OF PHYSIC, SURGERY, AND MEDICAL PHILOSOPHY.

*The Anatomy and Surgical Treatment of Inguinal and Congenital Hernia, illustrated by plates; by* ASTLEY COOPER, F. R. S. Member of the Royal Medical and Physical Societies of Edinburgh, Lecturer on Anatomy and Surgery, and Surgeon to Guy's Hospital. Folio, pp. 60.

THOUGH the world in general is not quite aware of the extreme frequency of hernia, every medical man knows that this disease is one of constant occurrence in every rank and station of life, and the most limited practitioner must feel the necessity of studying a complaint which forms no inconsiderable share of the business of the surgeon.

Notwithstanding the valuable labours of many eminent men in illustrating this difficult disease (for taking into account all its varieties and anomalies it may well be considered as difficult) there was still wanting (in this country at least) a clear accurate history of all that minute anatomy has been able to discover, and sound surgery to practise in the knowledge and treatment of hernia. This deficiency, it appears to be the object of the present volume to supply; and the high reputation of the author, as an anatomical teacher and practical surgeon, induces us to take an early notice of this publication.

In the Preface, the plan of the work is explained to be purely practical; that is to say, it is meant not to be a history of the opinions and practice of all who have written on hernia, but a history of the disease itself, founded on extensive and personal observation. "I have almost uniformly, in the following work," Mr. C. observes, "avoided quoting the opinions of authors on this part of surgery. This I have done, certainly not from any wish to slight or undervalue the labours of some of the most excellent physiologists and practitioners that have adorned our profession, but because it did not form a part of my plan to give a history of this branch of surgery, and because I wished to confine myself to the very wide scene of observation afforded by the two noble institutions of St. Thomas's and Guy's Hospitals, and to that portion of the practice of this metropolis which I have been personally enabled to authenticate. I have therefore related no case, and given no remark, to the truth of which I cannot vouch; and for the same reason, the subjects of all the plates annexed to this volume are from preparations either in my own possession or in the Anatomical Museum at St. Thomas's Hospital."

The Author pays a just compliment to the correctness of the Anatomical Nomenclature lately proposed by Dr. Barclay of Edinburgh; at the same time, however, that he declines adopting it in a practical work like the present. Probably this has been the most prudent course, though the defects of the present nomenclature are very striking in the description of relative situation, and the author is sufficiently aware of it by attempting something systematical in the employment of the terms at present in use.

The first Chapter contains a description of hernia in general, and particularly of the formation of the hernial sac, a subject which has excited some controversy, and has produced much nice and acute anatomical investigation, as it forms the most characteristic feature of the disease.

Few are so ignorant at the present day, as not to know that the antient term *rupture* or *bursten* is founded on an erroneous idea of the complaint making an opening or laceration of the parietes of the abdomen, and that the sac is formed by a gradual elongation of the peritonæum following the course of the spermatic cord; but there are circumstances in the density of the sac, adhesions, and the like, which are to be explained by more accurate anatomy. Sometimes the sac is wanting, the reason of which Mr. C. explains to be, either where the force of protrusion proceeds beyond the point at which elongation stops, to that where absorption begins; or where the hernia occurs at preternatural openings through the abdomen in the parts of this cavity not lined by peritonæum.

The second Chapter is a short, condensed, but admirable sketch of the anatomy of the parts connected with inguinal hernia. It is particularly here that (as was observed in the Preface) care is taken to preserve uniformity in the terms of relative situation commonly employed, and the whole is drawn up in the very clearest and most perspicuous manner. We rather wish that the Author had given some precise name to the *upper opening* from the abdomen at which the hernia first quits this cavity, before it takes its oblique downward course along with the spermatic cord to the abdominal ring. It is now sufficiently known, and has been taught in the Anatomical Schools for many years, that the passage of the spermatic cord out of the abdomen is not immediately opposite to the ring, but higher up towards the superior spinous process of the ilium, and this orifice ought to have had some designation as well as the abdominal ring.

The description of the fasciæ given off by Poupart's ligament, or, as Mr. Gimbernat terms it, the *crural arch*, is very minute. Mr. C. mentions two, more peculiarly concerned in the formation of hernia; one of them is noticed by Gimbernat, the other is here made the object of particular attention. It is sent upwards from the crural crural arch, immediately behind all the abdominal muscles, furnishing them with a tendinous expansion, and passes on each side of the spermatic cord, leaving a hole for its passage. It appears more than any other to regulate the size of the upper abdominal opening, and to be the specific contrivance which Nature has prepared for supporting the contents of the belly when protruding against this weak part of its parietes. The course of the arteries connected with hernia, and particularly the epigastric, is described with a minuteness adequate to the importance of the subject, where a want of knowledge in the operator will expose the life of his patient to imminent risk.

In the third Chapter, Inguinal Hernia is particularly described, both from its outward form and the appearances on dissection. The gradual approach made by the pressure of large herniæ of the upper abdominal opening towards the ring is exhibited, and some of the rarer varieties of situation relative to the spermatic cord, or a part of its contents. Of the diseases with which Hernia is liable to be confounded, the Varicocele appears to be that which is the most perplexing, we shall therefore give the Author's diagnosis.

"But of all the diseases of the scrotum which are ever mistaken for hernia, none is so much so as the Varicocele. Often have I known persons (even the children of medical men) to wear trusses for a supposed hernia, which they complained did not fit, gave them pain, and could not prevent the descent of the tumour, when it was found that the disease was the enlargement of the spermatic veins. Varicocele has indeed many marks of hernia. When large, it dilates upon coughing, but not otherwise; it appears in the erect posture, and retires when the body is recumbent; and it is first observed near the ring. The only sure method of distinction with which I am acquainted is this: Place the patient in the horizontal posture, and empty the swelling by pressure upon the scrotum; then putting the fingers firmly upon the upper part of the abdominal ring, desire the patient to rise. If it is a hernia, the tumour cannot re-appear as long as the pressure is continued at the ring; but if a varicocele, the swelling returns with increased size, owing to the return of blood into the abdomen being prevented by the pressure. Some judgment may also be formed by the feel of the tumour, for that of varicocele is always ropy, as if a bundle of cords were contained within the scrotum."

The causes of Inguinal Hernia are described in the fourth chapter.

Many sound and good remarks are introduced in the fifth chapter, on the use and application of trusses in the reducible hernia. The precise part of pressure is determined by the sure guide of Anatomy to be, not at the ring, but higher up, opposite the upper opening, more approaching the ring however in large and old herniæ than in the small and recent.

Irreducible hernia is next described, and some valuable cases introduced in illustration. The use of the laced bag truss is strongly recommended, where the return is absolutely impracticable.

The

The symptoms of strangulated Hernia are given in the next and very important Chapter, with that minuteness which is only the result of long experience and accurate attention. The seat of strangulation is the point to which the chief attention is always to be directed. The Author finds it to be sometimes at the ring, but much oftener above it, at the upper opening into the abdomen. The supposed *spasmodic* nature of the stricture is thus ingeniously illustrated. The ring itself being tendinous, cannot assume the state of spasm ; " but when the strangulation is above the ring, a portion of intestine protrudes under the edge of the internal oblique and transversalis muscles, compressing them, which in their turn being excited to contraction by the irritation of this pressure, re-act upon the intestine with a force sufficient to produce a strangulation accompanied with spasmodic symptoms."

The thickening of the mouth of the sac, Mr. C. finds by dissection to be a rare occurrence.

The treatment of strangulated hernia and the means for reduction by the *taxis*, or without the use of the knife, are given in the next chapter. The whole is purely practical, and has every evidence of being founded on very extensive observation. It may be considered as a fair specimen of the surgery of a considerable part of this metropolis. We shall not enumerate the various applications auxiliary to the taxis, but remark upon one or two of them.

In speaking of that powerful remedy the tobacco glyster, the author gives a caution which ought not to be forgotten. The dose here prescribed is only *one drachm* of tobacco infused for ten minutes in twelve ounces of boiling water; and he adds, " to those who have commonly heard of two drachms being thrown up at a time without bad consequences, this may appear an useless precaution ; but, instructed by personal observation, I can venture to assert, that whoever practises this often, will meet with effects which will lead him to repent his rashness.'

" I once saw a man with whom the tobacco glyster had been used in the quantity of two drachms without a reduction of the tumour, who about half an hour afterwards was put upon a table to have the operation performed, when his pulse was found so low, his countenance so depressed, and his body covered with cold sweats, that he was ordered back to bed, and on carrying him thither he expired."

Another case is added, in which *only one drachm* of the tobacco was used, which produced violent pain, vomiting of matter smelling strongly of tobacco, and terminated fatally about half an hour afterwards, evidently from the effects of the remedy.

An useful precaution is given on the application of ice, which is, not to let the part be moistened with the melting ice, as a long application of cold accompanied with wet, has been found to produce a local mortification or frost-biting of the integuments. This happened to a patient of Mr. Sharp and Mr. Cline. The ice should therefore be tied up in a bladder.

[ To be continued. ]

*Observations on Pulmonary Consumption; or, an Essay on the Lichen Islandicus, considered as an Aliment and a Medicine in that Disorder; by* J. B. Regnault, M. D. &c. Second edition. 8vo. pp. 112.

The first edition of this pamphlet was noticed in vol. viii. p. 463. Since that time the lamentable disease which is the subject of it, has engaged more general attention, and the means of prevention and cure have been more accurately considered. The remedy and treatment recommended by Dr. R. have been extensively tried, and in many instances with the desired success.

The present edition is considerably enlarged by the introduction of several new chapters; from the last of which we extract the following Observations, as they appear to merit the general attention of the public.

"It has long been the custom in England to send consumptive patients into warmer climates, such as the south of France, Spain, or Italy, for the sake of enjoying a drier and purer air. Without attempting to investigate the origin of this practice, let us enquire, in the first place, what proofs have been adduced in support of the opinion that a dry air is most favourable to the consumptive. Uniform experience shews us, that such invalids are much worse in a dry serene air, such as generally accompanies an east wind in this country, while, on the contrary, they are much better in situations where the air is mild, and charged with humidity; such as the south of England, or the sea, when the wind is in the south or west quarters. The function of the lungs requires perpetual and considerable motion, or expansion and contraction, and their flexibility or suppleness is supported by a thin aqueous mucus which lines all their cavities, and is secreted there for that purpose. It appears then, that an atmosphere charged with a certain degree of moisture is friendly to the lungs; and that it is a powerful means of preventing the inflammation which would destroy their motion, as happens in pleurisy and in a hot dry air.

"The bodies of animals ought naturally to be in a kind of vapour bath, formed by an atmosphere of their own, which always contains humidity to the distance of some inches; an air too hot and dry dissipates this continually, and that the more easily as it is supplied very scantily on account of the exhausted state of the fluids of the body.

"In hot sandy climates, as Egypt for example, all the resources are employed which art can suggest to moderate the heat and supply the atmosphere with the necessary moisture. They bathe frequently; they make wells in the middle of their houses; they receive the cool air of grottos by means of tubes; they live in cellars in the hot weather; keep their windows open during the night, and close those carefully which are exposed to the sun, during the day; they sprinkle their apartments with fresh water, or spread leaves or flowers over them; thus the air becomes charged with the moisture ture that evaporates, by which its elasticity is diminished, and the parching dryness of it rendered supportable.

"Dr. Pugh has demonstrated, by arguments founded on experience, that the climate of Naples or Nice, is very inimical to consumptive people. He instances a great number of English who died in a short time in these towns, a large proportion of which would certainly have escaped if they had remained in England, or chosen other parts of the south of France. He then proceeds to enumerate certain cantons of France, which he prefers to any part of Italy. He mentions, for the winter season, the environs of Avignon, of Nimes, and of Pezenas; principally because these districts are at a suitable distance from the sea, the influence of which he thinks so prejudicial in cold weather.

"In summer, he prefers Bareges or Bagneres, both situated on mountains, even to the mountains of Cevennes, which should be left about November, in order to return to the proposed winter residence.

"Though we approve Dr. Pugh's opinion, we would ask, what occasion is there to leave England, since it presents the very advantages we go so far in search of, and is exempt from the inconveniences attending distant changes of place? If change of air, in an atmosphere most equal and least agitated by tempests, is advantageous to persons of weak or diseased lungs, we ought to chuse this atmosphere as near as possible to the country where such invalids have previously lived; for if it be too remote, they will be exposed to new dangers in the journey. The different temperature of distant climates, the different exhalations from land and water, from animals, vegetables and minerals, have a powerful influence on different constitutions, and on health in general.

"To these important considerations, which are sufficient to determine our voluntary choice of situations for invalids as near their own homes as possible, we may now add the imperious motive of necessity. An ambitious enemy has shut the English out from France, Italy, and Spain, so that now they ought to do what reason has long suggested to them, that is, to seek for and prepare suitable situations for invalids, in their own islands. Bath and the hot-wells of Bristol have long been resorted to by asthmatic and consumptive patients; but the mild and sheltered vales of Devonshire and part of Cornwall offer situations far more desirable. It is a consideration of no light importance, that those who are obliged to leave their families for the recovery of health, may easily correspond with or be visited by their parents, children, or relations, and on many occasions be attended by them. The want of this convenience, together with the dangers and expences of distant travelling, have proved fatal to many a valuable life, by deterring patients and their friends from encountering long journies. Nothing seems necessary to draw these situations into general notice, but the patronage of a few noble and respectable families, to encourage the building of hotels, baths, promenades, and making good roads to to the adjacent towns. A small part of the sums heretofore expended in visiting the continent would be sufficient to secure all the salutary advantages of foreign climes on the English shores, without risking the dangers of voyages and travels. The mildness of the air on the south-western coast of England, Wales, and Ireland, is abundantly proved by the myrtles and other tender plants resisting the usual winters without artificial warmth. And surely it must strike every patriotic mind, that retaining the wealth of the nation, or distributing it in the country, is infinitely preferable to dissipating it among foreigners. We hope these observations, which are no less dictated by prudence than necessity, will promote inquiries and exertions, which cannot fail to improve the districts where they are made, as well as contribute to the general health and prosperity of the British empire."

